# Mammography screening in less developed countries

**DOI:** 10.1186/s40064-015-1394-8

**Published:** 2015-10-15

**Authors:** JunJie Li, ZhiMin Shao

**Affiliations:** Department of Breast Surgery, Shanghai Cancer Center and Cancer Institute, Shanghai Medical College, Fudan University, Shanghai, People’s Republic of China

**Keywords:** Breast cancer, Mammography screening, Less developed country

## Abstract

Less developed countries (LDCs) are struggling with an increasing burden of breast cancer. It is important to identify what interventions might be most effective and feasible in reducing overall breast cancer mortality in a resource constrained settings. Mammography screening (MS) utilized in developed countries cannot be equally applied to LDCs. We provide a summary of the status of existing and past MS program attempts in LDCs, and try to determine the prerequisites under which any developing country is ready to benefit from a MS program. We make the case for a “mixed” portfolio of tools to reduce breast cancer mortality with MS reserved only for those sub-populations that meet the criteria. We hope our review will provide a background for policy makers to apply rigorous criteria before attempting to implement costly MS program and before judiciously evaluating additional competed programs in their countries.

## Background

Since 2008, breast cancer incidence has increased by more than 20 % and mortality has risen by 14 % (The International Agency for Research on Cancer (IARC) 2013). In 2012 1.67 million new cancer cases and 0.52 million cancer-related deaths were reported (Ferlay et al. 2013). Of these, 52.9 % of global cases and 62.1 % of breast cancer deaths occurred in less developed countries (LDCs) (CIA-The World Factbook 2014). Breast cancer mortality-to incidence (M/I) ratio for less developed regions is 0.37, compared with 0.20 for more developed regions (Ferlay et al. 2013). Health ministries and health-care systems in LDCs are struggling to respond to the increasing morbidity and mortality from advanced cancers among patients treated in the public system and among the urban poor, rural, remote and indigenous populations (Goss et al. 2013). Several studies have described the current status and challenges of breast cancer facing a number of key LDCs including Brazil, (Lee et al. 2012) Mexico (Chavarri-Guerra et al. 2012) and China (Fan et al. 2014). For all of these countries, mortality is much higher due to low cancer awareness, lack of early detection with concomitant higher stage of disease at presentation, lack of an implemented, effective national screening program and lack of timely access to optimal cancer care, all of which challenges the ability of these LDCs to ameliorate their cancer burden.

Stated by the International Agency for Research on Cancer (IARC) that “an urgent need in cancer control today is to develop effective and affordable approaches to the early detection, diagnosis, and treatment of breast cancer among women living in LDCs” (The International Agency for Research on Cancer (IARC) 2013). Patients with late stage breast cancer associated with more substantial economic burden even in developed countries (DCs), (Remak and Brazil 2004) we believe developing an effective and affordable early detection approach may be the first step for majority LDCs.

An effective mammographic screening (MS) program is considered the best tool available for early stage detection of breast cancer and can potentially reduce the risk of death from breast cancer (Wubker 2013; Apesteguia Ciriza and Pina Insausti 2013). However, it has not been without controversy, views of the possible risk:benefit of MS are sharply polarized and increasingly vocal (Marmot 2013). In 2002, the IARC thoroughly reassessed evidence on the effectiveness of MS with a goal of interpreting effectiveness and to attempt to balance expected risk:benefit ratios in specific target populations, aim to formulate recommendations for further research and public health action (The International Agency for Research on Cancer (IARC) 2002). Until now the debate has still not been resolved. Some countries such as the United States (US) (US Preventive Services Task Force 2009) and Canada (Tonelli et al. 2011) have modified or updated their recommendation for MS programs timely. Likewise the United Kingdom (UK), has recently convened an independent panel to review the evidence (Marmot et al. 2012). It is apparent that universal standards for the implementation of MS programs in different countries, and within a specific country is not feasible, due to enormous variations in the population and availability of resources, and differing interpretations of the existing data. This has led to a wide diversity of recommended guidelines (Table 1).

**Table 1:** Mammography screening programs in selected LDCs and DCs

Country	Organizational level	Year implemented (nationwide)	Participation rate	Interval (years)	Screening age
LDCs					
Russia	Khanty-Mansiysky autonomous Region-Yugra	2007	67.50 %	2	>40
Brazil	State of Sao Paulo	2003	56.70 %	2	40–69
Mexico	Mexico City	2005	50 %	2	40–69
Uruguay	Nationwide	2006	Mandatory	2	40–59
Hungary	Nationwide	2002	56.30 %	2	45–65
Croatia	Nationwide	2006	60 %	2	50–69
Poland	Nationwide	2007	40 %	2	50–69
DCs					
Sweden	Nationwide	1986 (97)	81 %	1.5/2	40–74
UK	Nationwide	1988 (96)	76 %	3	50–64
Canada	Nationwide	1988	79 %	2/3	50–74
US	Nationwide	1991	83 %	1	≥40
US-ACS				1	≥40
US-ACR				1	≥40
US-ACOG				1	≥40
USPSTF				2	50–75

*LDCs* less developed countries, *DCs* developed countries, *ACS* American Cancer Society 2003, *ACR* American College of Radiology 2013, *ACOG* The American College of Obstetricians and Gynecologists 2011, *USPSTF* US Preventive Services Task Force (2009)

Whether MS programs should be implemented or not for the goal of down-staging and reduce mortality of breast cancer in LDCs is challenging the notion. Based on their current resource-limited health-care systems, most LDCs believe that “raising breast awareness may be a priority goal, before trying to implement wide spread population-based screening” (Harford 2011). However many LDCs are undergoing significant economic growth and change and have rapidly changing socioeconomic, infrastructure and ethnic changes occurring. It is thus an increasing challenge for all LDCs to gain an understanding of the specific circumstances and criteria in which they would consider launching a MS program. Herein we provide a summary of the status of existing and past MS program attempts in LDCs and try to provide criteria on which this decision can be based.

## Methods: search strategy and selection criteria

Medline (OVID), Pubmed and Web of Knowledge databases were searched for the subject headings: “breast neoplasm,” “breast cancer,” “breast,” and “screening” both as single headings and combined, up to December 2013. Reference lists from relevant individual articles were examined for additional relevant articles. Full papers and/or abstracts were reviewed and included to illustrate the current status of mammography screening in LDCs if: (1) Mammography screening and related topics were specifically the topic of the manuscript or emphasized within it; (2) breast cancer screening described in the article referred specifically to women living in LDCs (not including those who live in or immigrated to developed countries); (3) the article (abstract) was published in English between 2002 and December 2013 [literature published subsequent to the published IARC Handbook (The International Agency for Research on Cancer (IARC) 2002) of breast cancer screening in 2002].

## Review

### The current status of mammography screening in LDCs

**Fig. 1:**
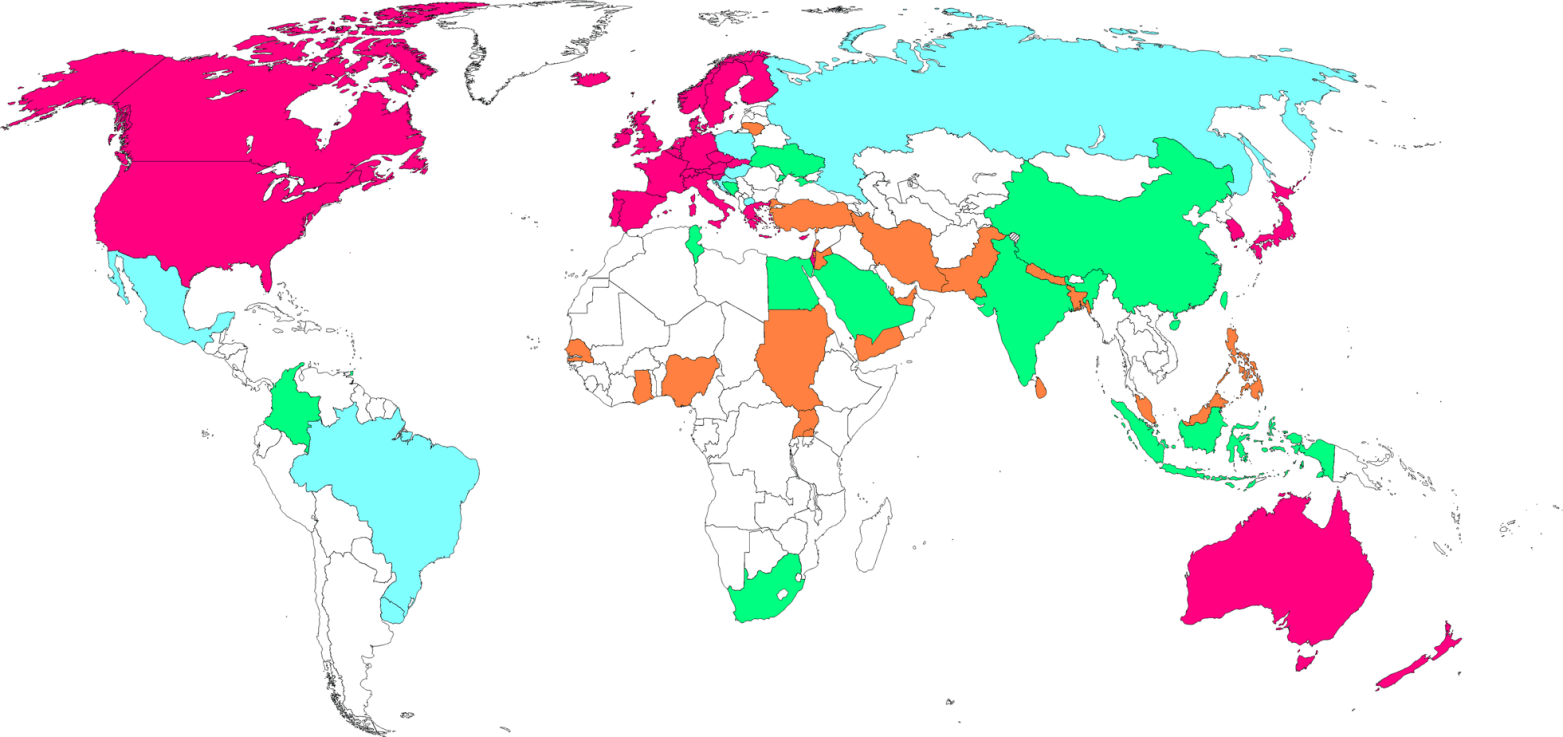
Current Status of mammography screening in LDCs. (All developed countries are shaded in *red*). Level I refers to countries which have nationwide or localized mammography screening programs (shaded in *blue*: Russia, Brazil, Mexico, Uruguay, Hungary, Croatia, Poland and Macedonia); Level II refers to countries which have trials or studies in particular populations for the evaluation of mammography screening accuracy or cost-effectiveness (shaded in *green*: South Africa, China, India, Indonesia, Tunisia, Trinidad and Tobago, Bosnia and Herzegovina, Colombia, Ukraine, Saudi Arabia and Egypt); Level III refers to countries which have surveys or questionnaires on breast cancer screening awareness and access to mammography (shaded in *orange*: Sudan, Nigeria, United Arab Emirates, Turkey, Jordan, Iran, Ghana, Pakistan, Bangladesh, Malaysia, Sri Lanka, Uganda, Lebanon, Senegal, Nepal, Philippines, Lithuania, Palestine, Yemen and Qatar); Level IV refers to countries with no data reported (shaded in *white*)

Through a literature search, we retrieved 246 articles relevant to MS in 40 LDCs. We divided LDCs into four levels of readiness for MS program implementation according to the articles we retrieved, as shown in Fig. 1.

### Level I: LDCs with evaluable MS programs, nationwide or localized region

Eight LDCs have published results from their nationwide or local MS programs (Fig. 1 shaded in blue). Table 1 compares the relevant criteria used to base the MS program on and the MS guidelines that were in place at the time of the programs. In Russia, (Zakharova 2013) Brazil, (Barreto et al. 2012; Mauad et al. 2011) and Mexico, (Rodriguez-Cuevas et al. 2009) due to large populations and diverse health care systems, MS is delivered in selected localized regions of the countries only, rather than a national MS program. In contrast, Hungary, (Boncz et al. 2013) Croatia, (Stamenic and Strnad 2011) Macedonia (Gershan and Antevska-Grujoska 2011) and Poland (Matkowski and Szynglarewicz 2011) has allowed implementation of nationwide MS programs. Since 2006 Uruguay has a unique mandatory screening program termed “the obligation for working women” (female employees must complete breast screening in order to obtain a “health card” that all workers must have (Arie 2013). Recommended screening age in Russia, Brazil, Mexico, Uruguay and Hungary includes women from age 40 and above, whereas in Croatia and Poland, screening is initiated in women only from age 50. Based upon the available data most countries set an upper age of 69 years, although in Brazil no upper age limit is defined. Screening is generally conducted biennially. Adherence rates vary considerably, it is noteworthy that all programs have adherence rates well below 70 %, a level that is considered necessary to confer a reduction in breast cancer mortality (WHO 2008).

### Level II: countries which have conducted studies and/or trials to evaluate the accuracy or cost effectiveness of a MS program

Twelve countries have published pilot studies (Fig. 1 shaded in green), consisting of several sequential MS within a specific relatively resource-rich area within their country. These studies have evaluated several important metrics: overall breast cancer detection rates; early detection rates; difficulties in implementation; strategies to expand adherence rates and; cost analyses aimed at informing policy makers on the importance and feasibility of a nationwide organized MS program. One example is a study from India which found CBE performed annually from age 40 to 60 almost as effective as biennial MS in terms of reduction of breast cancer mortality, while incurring only half the total cost of a MS program, (Okonkwo et al. 2008) suggested that western MS programs may not be cost-effective in LDCs, especially in light of competing health care priorities and economic conditions.

### Level III: countries with population-based surveys or questionnaires on breast cancer screening awareness and access to opportunistic mammography

Twenty LDCs (Fig. 1 shaded in orange) have no published studies or trials of MS in their countries. However, population-based surveys of public breast cancer awareness and opportunistic MS have occasionally been conducted. Generally, women in these LDCs were shown to have low breast cancer awareness, and mammography utilization rates were very low. Only 18.75 % of female physicians and 17.24 % of female non-health care personnel had mammogram in Iran (Kadivar et al. 2012). One survey in a very low resource area of South Africa reported that “no woman had ever had a mammogram” (Maree et al. 2013).

### Level IV: countries with no MS data reported

The remaining LDCs (shaded in white) have not reported any study related to the use of MS.

We also found that varying recommendations and/or guidelines related to mammography are frequently issued by governments in LDCs but with no involvement of ministries of health and no plan for guaranteeing implementation or monitoring of quality control or compliance. Up to now, opportunistic MS in some countries such as Brazil and Mexico do not have any information or databases to understand what has been really going on. Some countries have voiced the intent to launch preventative “screening mammography” programs, but in reality they frequently are referring to “diagnostic mammogram programs”, frequently involving a mammogram either after an opportunistic mammogram has been read as suspicious or after women have experienced symptoms suspicious of breast cancer (National Breast Cancer Foundation 2014). Thus inappropriate attempts to implement such a program in a resource constrained country may lead to disappointing results and contentious use of health care budgets (Tiezzi 2013).

There are no existing methods to evaluate whether a LDC is qualified to have a MS program, Breast Health Global Initiative (BHGI) has proposed stratified guidelines for early detection in resource constrained countries and true MS programs be reserved for “enhanced” and “maximal” resourced countries (Anderson et al. 2011; Yip et al. 2008). In this manuscript we summarize certain prerequisites which must be met for a LDC to consider launching a MS program with a view to reducing national breast cancer specific mortality.

### Criteria required for implementation of a MS program in LDCs

Here we propose five steps to determine readiness for a MS program (Table 2) as followed:

**Table 2:** Criteria required for implementation of a mammography screening program in LDCs

Target population	Breast cancer prevalence, incidence and mortality rates
	Life expectancy
	Sensitivity and specificity to mammography
	Socioeconomic and educational level
Resources	Mammogram equipment (quantity and quality)
	Trained personnel
Program methods	Age of initiation (40 or 50)
	End age (69 or >70)
	Frequency (once every 1, 2, 3 years)
	View (single or double)
	Technique (digital or film)
	Review method (1 or 2 radiologists)
	Combine BSE and/or CBE
	Combine other image
Outcomes	Short term outcome
	Mortality deduction rate
	Overdiagnosis rate
	False Positive rate
Cost-effectiveness analysis	Methods of CEA
	Crosswise and longitudinal comparison

### Target population

The first step is defining an appropriate “target population,” a group who will have substantial net benefit from MS (Quanstrum and Hayward 2010). Target populations differ both between and within countries mainly depending on: breast cancer burden and life expectancy; sensitivity and specificity of mammography; socioeconomic and educational level.

Disease burden (breast cancer prevalence, incidence and mortality rates) and life expectancy MS can be successful when there are a critical number of potential breast cancer patients in the prevalence pool. There is a correlation between a country’s GDP and its age-standardized breast-cancer incidence, (Harford 2011) incidences of DCs are 2–4 times of incidences of LDCs (as shown in Table 3). However, the incidence has increased rapidly, especially in socioeconomic developed areas of LDCs. For example, the observed age-standardized incidence rates in the eastern coastal urban areas of China are similar to those observed in Japan (Fan et al. 2014). Of note, MS itself may contribute to the excess incidence rate in DCs (Welch and Black 2010). In the US female population age 50 and older, the age adjusted incidence of invasive breast cancer was around 273.86 (per 100,000 people) in 1975, which rapidly increased to almost 396.94 by 1999 (Howlader et al. 2013; DeSantis et al. 2014). Similar increases were also observed in the UK, Canada, Sweden and Norway, after MS implementation (Jorgensen and Gotzsche 2009). Hence, some areas in LDCs may already have enough potential patients and are qualified to have MS. Estimates for breast cancer incidence can be obtained from Globocan 2012, however, most LDCs cannot provide details on age-specific breast cancer prevalence and incidence rates, posing a challenge to accurately identify the best screening age group.

**Table 3:** Breast cancer burden and demographics of selected countries

	Incidence	Mortality	M/I ratio	5-year prevalence proportion per 100,000	Female population % of total	Population density people/km^2^	Life expectancy at birth, female years
China	22.1	5.4	0.24	129	1,350,695,000 (48.2 %)	144	76
India	25.8	12.7	0.49	92.6	1,236,686,732 (48.3 %)	411	68
Brazil	59.5	14.3	0.24	317.8	198,656,019 (50.8 %)	23	77
Russian Federation	45.6	17.2	0.38	328.3	143,533,000 (53.8 %)	9	75
Egypt	49.5	19.3	0.39	222.5	80,721,874 (49.8 %)	80	73
Sudan	27.8	15.2	0.55	108.8	37,195,349 (49.8 %)	20	63
US	92.9	14.9	0.16	753.7	313,914,040 (50.8 %)	34	81
UK	95	17.1	0.18	755.1	63,227,526 (50.8 %)	259	83
Canada	79.8	13.9	0.17	666.8	34,880,491 (50.4 %)	4	83

Incidence and mortality rate are defined as the age-standardized incidence or mortality per 100,000 people per year. Incidence, mortality, Mortality-to-incidence (M/I) ratio and 5-year prevalence proportion were obtained from Globocan 2012. Population, population density and life expectancy at birth were obtained from the World Bank data from 2012 (The World Bank 2014b)

No data explicit declared the minimum absolute mortality from breast cancer needed to warrant a MS program, however, the mortality rates of some LDCs are similar to those of DCs (Table 3). Female overall life expectancy is another parameter requiring consideration as low life expectancy results in fewer life years that can be saved via MS. For example, in Sudan the breast cancer mortality rate (15.2 per 100,000 people) is similar to that of the US (14.9) and Canada (13.9), however Sudanese female life expectancy is almost 20 years shorter. Thus even if Sudan has met the other qualifying prerequisites, the population as a whole would not benefit from MS.

#### Factors effecting sensitivity and specificity of MS

Mammographic sensitivity was 80 % among women with predominantly fatty breasts, and just 30 % in women with extremely dense breasts (Mandelson et al. 2000). With age increases, breast density decreases and MS efficacy increases. MS reduces mortality by about 20–35 % in women aged 50–69 years, with less reduction in women aged 40–49 years, (Elmore et al. 2005) and increased false positive in younger women with high density (Salas et al. 2011). Hence, target population with low breast density may benefit more from MS. Breast density may exist across racial groups, greater in Asian women and least in African women, rely on BMI, age at screening, diet and alcohol intake, (del Carmen et al. 2007; Voevodina et al. 2013) differences in age-specific breast density and mammographic sensitivity need to be considered when deciding whether to initiate MS in any specific LDC.

#### Socioeconomic and educational level

Socioeconomic and educational levels and cancer awareness all affect the feasibility and utility of MS programs. Women in the US and Canada with high incomes and education levels were more likely to receive MS, (Katz and Hofer 1994) and women of low socioeconomic status show a low re-attendance screening rate according to the experience of Ontario Breast Screening Program (Tatla et al. 2003). Population in many LDCs is of insufficient socioeconomic status and low cancer awareness, which will make a screening program of insufficient utility, (Goss et al. 2014) such as the coverage rate of one regional MS program was shown to be is only about 30 % in Russia (Zakharova et al. 2011). Hence, health care systems must have medical record systems that follow patients over time, and education center may help to increase re-screening rate. For example, through telephone reminder and guidance, percentage of women had mammograms increased from 3.9 to 46.4 % in Turkey (Baysal and Gozum 2011).

Due to the points discussed above, simply applying the screening population taken from the existing guidelines from DCs will not be effective in LDCs. In LDCs, the average age of women with breast cancer is 10–20 years younger than women in the Western countries (Moore et al. 2003). Therefore, we suggest that women with the highest breast cancer incidence rate in socioeconomically well-developed areas of LDCs can attempt to be considered for the target population of a MS program. The potential size of the target population can be estimated with age distribution data.

### Evaluation of mammography resources and requirements for a MS program

A minimum density of mammography machines is necessary in order to facilitate an efficient and accessible nationwide program. Most LDCs lack adequate health care infrastructure for their entire population, (Melnikow et al. 2013) the ratio of mammography equipments to physicians who perform CBE is close to 20 % in the US, but less than 0.25 % in India, (Sarvazyan et al. 2008) and in 2010 Sudan had only nine mammogram machines (WHO 2010. Canada has a mammography machine density of 72 per 1,000,000, while Mexico has a density of 37 per 1,000,000 (Chavarri-Guerra et al. 2012). The distribution of equipment is irregular due to economical heterogeneity in LDCs. In Brazil, the distribution ranges from two mammography machines in the northern state of Roraima (population of 450,579 in 2010) to 335 machines in the southeastern state of São Paulo (population 41,262,199 in 2010) (Lee et al. 2012). The distance the target population must travel and how long it takes to access MS may impact the adherence rate of a program. Whether mobile mammography, which may help women overcome the socioeconomic barriers to screening in US, such as lack of transportation, financial limitations or lack of medical insurance, (Carkaci et al. 2013) can be applied in LDCs with low population density need further investigation.

The quality and maintenance of mammography equipment is also of importance, as an example 20 % of mammography facilities in some areas of Brazil are out of use (Lee et al. 2012). Meanwhile, competency and continuous medical education of personnel working in a MS program also needs attention. A scarcity of technicians and radiologists specialized in breast imaging in Mexico and in the majority of Latin American countries, (Rodriguez-Cuevas et al. 2009) may lead to a sub-optimized outcomes of MS implementation in these resource limited LDCs.

### Optimizing methods of MS program

How to optimize and form an “effective and affordable” method for the implementation of MS in LDCs is a big challenge.

#### Screening methods: age range, interval, direction, technique and review methods

Screening strategies differ in the existing and past MS programs (shown in Table 1). No evidence showed different methods led to different screening outcome in LDCs. Data from DCs demonstrate that screening benefit is reduced in younger women, even with shorter intervals (Pace and Keating 2014). Hence, although some guidelines recommend initiating annually mammography at age 40, (Smith et al. 2003; Henderson et al. 2003; American College of Obstetricians-Gynecologists 2011; Drukteinis et al. 2013) the US Preventive Services Task Force (USPSTF) recommends against routine screening mammography in women aged 40–49 years, and biennial screening mammography for women between the ages of 50 and 74 years (US Preventive Services Task Force 2009). For women over 50 years in UK, annual screening is predicted to have a relatively small effect on breast cancer mortality, with a relative risk of death at 0.95 compared with a 3-year screening interval (United Kingdom Co-ordinating Committee on Cancer Research 2002). Single-view mammography was used in the Swedish Two-county and Stockholm trials, while two-view mammography was used in the Malmo, Goteborg, Health Insurance Plan and Canadian trials, and in the first screen in Edinburgh, (The International Agency for Research on Cancer (IARC) 2002 two views were medically more effective and reduced recall rates, (Wald et al. 1995) while no significant difference found in the reduction in breast cancer mortality between one view or two views (Kerlikowske et al. 1995). No randomized controlled trials have compared digital or film mammography specifically, and although digital mammography is generally considered to be more effective it is more expensive (Melnikow et al. 2013). Double reading by two physicians increased the rate of cancer detection, (Dinnes et al. 2001) although using computer-aided detection with final diagnosis determined by one physician might be more cost-effective method (Sato et al. 2012).

#### Combining CBE/BSE (breast self exam) or other imaging technology

The evidence supporting the value of combining CBE and BSE as methods to reduce breast cancer mortality is limited and mostly inferential (Drukteinis et al. 2013). Canadian guidelines recommend against BSE and CBE; (Tonelli et al. 2011) USPSTF recommends against BSE and cites insufficient evidence to recommend CBE; (US Preventive Services Task Force 2009) and ACOG recommends annual CBE to women >40 years and BSE only for high risk patients (American College of Obstetricians-Gynecologists 2011). Ultrasound is an established adjunct to mammography in imaging evaluation, can be an option for additional screening in women at high risk, similarly in candidates for magnetic resonance imaging (Smith et al. 2003; Henderson et al. 2003). With routinely MS, CBE/BSE may not improve outcome, however, in LDCs, training of BSE by increasing cancer awareness, may also improve early detection. Limited evidence showed that women who had CBE were more likely to undergo MS in Malaysia, (Dahlui et al. 2012) and ultrasound combined with mammography may result in effective protection for Chinese women based on the younger age at tumor onset (Huang et al. 2001).

The best method is still inconclusive and data from studies performed in DCs should be considered prudentially in the setting of LDCs. It is necessary to create an “individualized” MS method for LDCs depending on different populations and medical resources, learning from the experiences in DCs and making appropriate improvements and modifications.

### Screening outcome

Short term outcomes of MS implementation appear promising in LDCs, in Brazil patients with clinical stage 0–1 disease increased from 50 to 70.8 %; (Silva et al. 2013). In Russia, the average cancer detection rate was 2.5 per 1000 women screened, with a significant increase in breast cancer incidence and a significant trend of decreased breast-cancer-related mortality; (Zakharova et al. 2011). In Hungary, percentage of women undergoing mammography increased from 27.4 to 61.0 % in 2002–2003, and 56.3 % in 2004–2005 after the introduction of the nationwide program (Boncz et al. 2008).

Long term outcomes are still unclear in LDCs, such as the precise mortality reduction rate, the overdiagnosis rate and false positive rate. Based on the current evidence from DC, they are still controversial (Jorgensen 2013). Some studies show a “significant” mortality reduction benefit of as much as 28–65 %, (Berry et al. 2005) while others show a “minimum” benefit of as little as 15 % (Gotzsche and Jorgensen 2013). Improvement in systemic treatments also contributed to mortality reduction, as 30 % reduction in breast cancer mortality was observed after screening and adjuvant therapy, 10 % of this reduction is due to screening and 20 % is due to treatment (Berry 2013). Overdiagnosis refers to MS detected breast cancers that might never have progressed to become symptomatic during a woman’s lifetime (The International Agency for Research on Cancer (IARC) 2002). SEER (Surveillance, Epidemiology, and End Results) incidence and mortality data showed increased cancer diagnosis with no obvious change in cancer mortality, suggesting the existence of overdiagnosis (Welch and Black 2010). Based on varying assumptions and statistical methods, one study extracted incidence data from two randomized controlled trials and found a “minimum rate” to be around 1 %; (Duffy et al. 2005) while a review based on published trends on incidence in five DCs found a “substantial rate” of 52 % (Jorgensen and Gotzsche 2009). Plausible estimates need appropriate adjustments for lead time bias and the pre-existing trend of increasing incidence in the population, so an overdiagnosis rate range from 1 to 10 % is generally accepted (Puliti et al. 2012). False positive findings are another inevitable challenge, may result in subsequent unnecessary surgical intervention, as well as greater short-term and long-term negative psychosocial consequences (Brodersen and Siersma 2013). Estimated false positive rate was 17 % for women undergoing 10 biennial screening tests in Europe, (Paci 2012) and about 61 % for 40- or 50-year-old women undergoing 10 years of annual mammography in US (Pace and Keating 2014).

There is no single gold standard for reaching an unbiased and reliable estimate of these long term outcomes, largely depending on the characteristic of target population and screening methods. Hence, we recommend small-scale MS trial should be conducted and followed up before the implementation of a nationwide program in LDCs.

### Cost-effectiveness analysis

All the parameters discussed above, including target population, screening resources, detail screening methods and evaluated outcome should be integrated into a CEA, which is the final key step to enable policy makers to put forward a medical practice (Weinstein and Stason 1977). Generally incremental cost effectiveness ratio (ICER) is accepted to estimate the expected cost of a MS to units such as per life year saved, per quality adjusted life year (QALY) saved, per disability adjusted life year (DALY) averted and so on (Laxminarayan et al. 2006). Biennial digital mammography beginning at age 50 resulted in an incremental cost per life year of US$17,050 in the US, (Melnikow et al. 2013) and once every 3 years beginning at age 50 in the UK was associated with £20,800 (US$34,784) per QALY gained (Pharoah et al. 2013). These results cannot be directly applicable to LDCs, each CEA should be assessed on a country-by-country basis. There is no data shows exactly what amount of “incremental cost” is acceptable for individual LDCs, definitely not as much as that identical to the £20,000–30,000 per QALY established in the UK (National Institute for Health and Care Excellence 2014). Complex models require many assumptions, usually estimated by extrapolating data from clinical results, are highly susceptible to both error and introduction of bias, (Detsky and Laupacis 2007) hampering CEA conducted in LDCs. Limited data demonstrated that biennial MS for women aged 40–60 years in India resulted in a cost of around US$3468 per life year gained, (Okonkwo et al. 2008) and around US$6516 (R$13573.07) in Brazil, (Peregrino et al. 2012) policy makers struggle with determining the net benefit and dare not to quickly implement such a program.

We suggest crosswise and longitudinal comparisons can be used to roughly estimate the practicality of MS in LDCs. In a crosswise comparison, for example, 1 year of adjuvant trastuzumab was determined to be a cost-effective treatment option in early-stage breast cancer, with the ICER per QALY was around US$8000 in China, (Chen et al. 2009) and US$18,970 in the US (Liberato et al. 2007). Based on ICER results of trastuzumab, by comparing ICER of MS in the US with the result estimated for MS in China, we can roughly estimate whether such a program is worthy to carry out in China. In a longitudinal comparison, we can use of the cost-effectiveness (CE)/GDP per capita ratio, (Yoo et al. 2013) as well as CE/yearly health expenditures per capita ratio, (van Ineveld et al. 1993) to balance the differences among countries. If the CE/GDP per capita ratio is greater than 1, then MS can be deemed inefficient, as the cost per life year saved exceeds the per capita GDP. Of note, individual cost-effectiveness ratios are estimated for a single program, not for a group of programs vying for a fixed budget (Detsky and Laupacis 2007). To maximize health outcomes from a limited budget, policy makers still face the challenge of prudentially and equitably allocating resources across a defined number of competing needs (Detsky and Naglie 1990).

According to this “five steps” model proposed here, we have tested MS in Shanghai cautiously, trying to meet all the criteria required for implementation. Shanghai is a prosperous urban city of China, with relatively better medical resources. Cancer registry was set up since 1970’, and “Shanghai Cancer Report” is published by Shanghai Center of Disease Control yearly, with detail information of age specific cancer incidence, mortality and prevalence. We selected women with highest incidence rate (age of 45–69 years old) as target population. Two-view film mammography with annual screening was used in a localized area in Shanghai. Sensitivity of MS was tested in different age group, 65.4 % in age 45–59 years and 66.8 % in age 60–69 years (Mo et al. 2013). With adequate follow-up, long term outcome can be analyzed and CEA will be conducted in the near future. Then screening methods may necessarily be modified, combing with breast exam and/or ultrasound. Only when net benefit has been observed, we may implement such a program to all over Shanghai.

## Discussion

LDCs have experienced a marked increase in breast cancer burden, (Ferlay et al. 2013) with poor cancer awareness of the public, delayed diagnosis, and non-optimal and untimely treatment. Efforts are needed to improve patient outcomes (Goss et al. 2014). Most LDCs prioritize early detection of breast cancer, and some may be eager to achieve this through MS programs. The aim of this review is to illustrate the status of existing and past MS program attempts in LDCs and outline the challenge and limitations of MS in these countries.

There are some limitations to this review. Firstly, the definition of LDCs varied- our definition is similar to the World Bank’s term “developing economies,” which includes low- and middle-income economies, with gross national income (GNI) per capita of less than US$12,616 (The World Bank 2014a). Discrepancies exist, for example, although Russia is categorized as a high income country, it is still regarded as a developing country by the World Bank, and therefore was included in our analysis. Secondly, our review was limited to literature published in English, therefore we may have omitted studies published in local journals or listed in non-English databases. Thirdly, our review does not cover all parameters related to launching a MS program, but instead we have selected factors which we believe are most important for defining a target population, assessing whether a country is well-equipped, and designing and evaluating the practicality of such a program.

Increasing efforts for breast cancer screening have been made in LDCs: national recommendations and/or guidelines have been laid out, MS programs had been incorporated in some countries and relevant studies have been conducted in others. Based on our review, there may be a role in LDCs to employ western style MS for targeted minority groups, such as high socioeconomic level and high risk populations. Meanwhile, for LDCs, it is also important to ensure patient follow-up to maintain high adherence rates, to ensure the accuracy of screening mammography, to shorten the waiting time to definitive diagnostic procedure after suspicious results, and to deliver state-of-the art treatment after early detection by screening. MS programs are currently too resource-demanding for most LDCs, due to the lack of breast cancer awareness among the population, the inadequate identification of appropriate target populations, the low likelihood of up-take and compliance, the scarcity of mammography resources, unaffordable screening costs and overall sub-optimal outcomes in survival benefit.

## Conclusion

**Fig. 2:**
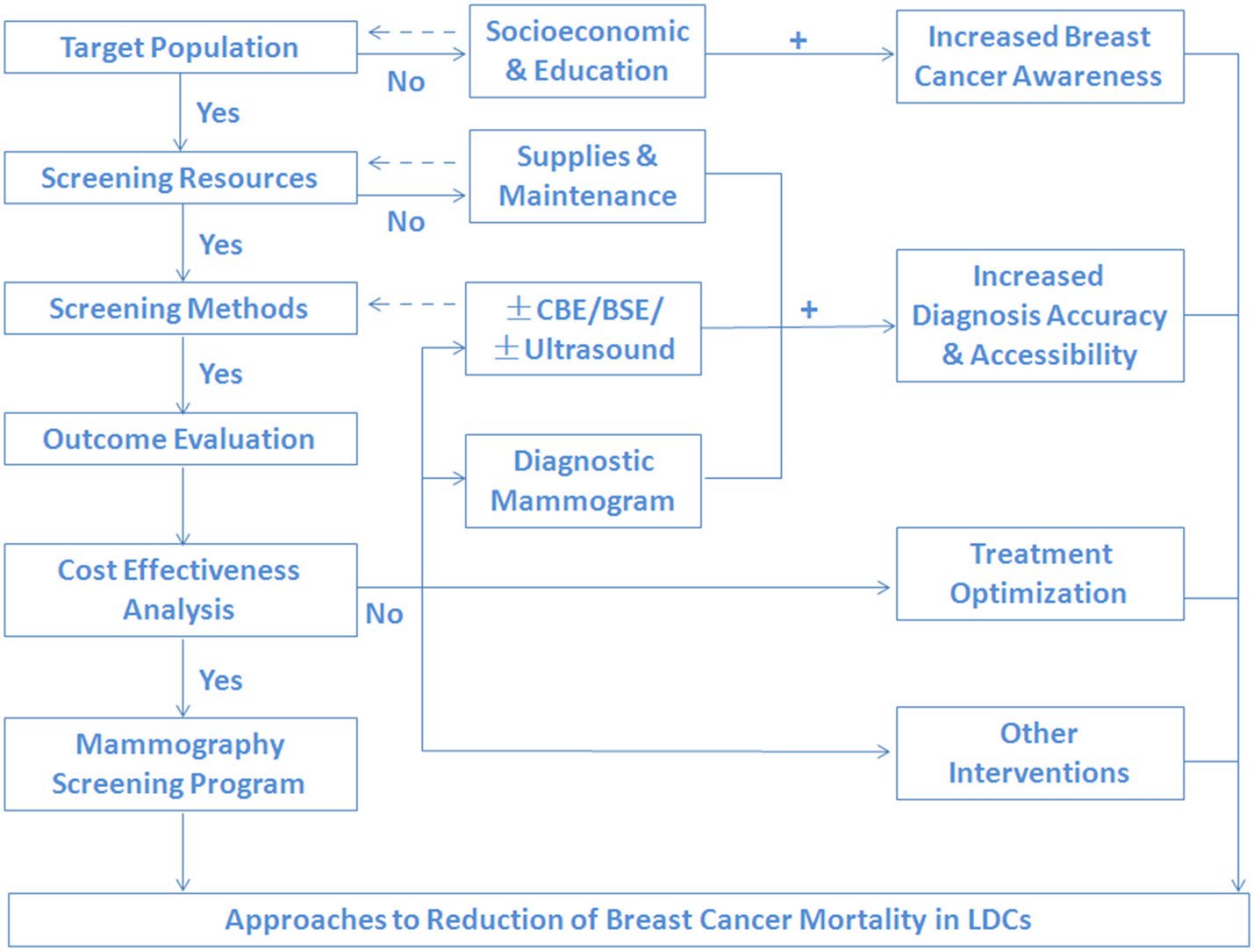
Approaches to Reduction of Breast Cancer Mortality in LDCs. The criteria for launching a MS program in LDC must be assessed in a stepwise fashion. If any of the criteria are not met, an organized MS program should not be pursued. Alternate strategies, such as cancer awareness improvement, modified screening methods or diagnostic mammography should instead be considered as the first line methods for reduction of breast cancer mortality

MS is one tool among many to promote early detection to downstage breast cancer diagnosis and mortality reduction. Improving the socioeconomic conditions and education level of the population are also methods which can drastically increase breast cancer awareness and improve the likelihood of improved adherence rates for a future MS programs implemented in LDCs. Overall, the best interventions for methods for mortality reduction of breast cancer must be customized to accommodate local conditions, and making the best use of limited resources is essential (Fig. 2: flow chart).

## References

[CR61] American College of Obstetricians-Gynecologists (2011). Practice bulletin no. 122: Breast cancer screening. Obstet Gynecol.

[CR1] Anderson BO, Cazap E, El Saghir NS, Yip CH, Khaled HM, Otero IV, Adebamowo CA, Badwe RA, Harford JB (2011). Optimisation of breast cancer management in low-resource and middle-resource countries: executive summary of the Breast Health Global Initiative consensus, 2010. Lancet Oncol.

[CR2] Apesteguia Ciriza L, Pina Insausti LJ (2013). Population-based breast cancer screening: certainties, controversies, and future perspectives. Radiologia.

[CR3] Arie S (2013). Uruguay’s mandatory breast cancer screening for working women aged 40–59 is challenged. BMJ.

[CR4] Barreto AS, Mendes MF, Thuler LC (2012). Evaluation of a strategy adopted to expand adherence to breast cancer screening in Brazilian Northeast. Revista brasileira de ginecologia e obstetricia: revista da Federacao Brasileira das Sociedades de Ginecologia e Obstetricia.

[CR5] Baysal HY, Gozum S (2011). Effects of health beliefs about mammography and breast cancer and telephone reminders on re-screening in Turkey. Asian Pac J Cancer Prev APJCP.

[CR7] Berry DA (2013). Breast cancer screening: controversy of impact. Breast.

[CR8] Berry DA, Cronin KA, Plevritis SK, Fryback DG, Clarke L, Zelen M, Mandelblatt JS, Yakovlev AY, Habbema JD, Feuer EJ (2005). Effect of screening and adjuvant therapy on mortality from breast cancer. N Engl J Med.

[CR9] Boncz I, Dobrossy L, Pentek Z, Kovacs A, Budai A, Vajda R, Sebestyen A (2013). Participation rates in the third round (2006–2007) of the breast cancer screening program in Hungary. Magy Onkol.

[CR10] Boncz I, Sebestyen A, Pinter I, Battyany I, Ember I (2008). The effect of an organized, nationwide breast cancer screening programme on non-organized mammography activities. J Med Screen.

[CR11] Brodersen J, Siersma VD (2013). Long-term psychosocial consequences of false-positive screening mammography. Ann Fam Med.

[CR12] Carkaci S, Geiser WR, Adrada BE, Marquez C, Whitman GJ (2013). How to establish a cost-effective mobile mammography program. AJR Am J Roentgenol.

[CR13] Chavarri-Guerra Y, Villarreal-Garza C, Liedke PE, Knaul F, Mohar A, Finkelstein DM, Goss PE (2012). Breast cancer in Mexico: a growing challenge to health and the health system. Lancet Oncol.

[CR14] Chen W, Jiang Z, Shao Z, Sun Q, Shen K (2009). An economic evaluation of adjuvant trastuzumab therapy in HER2-positive early breast cancer. Value Health J Int Soc Pharmacoecon Outcomes Res.

[CR15] CIA-The World Factbook (2014) https://www.cia.gov/library/publications/the-world-factbook/appendix/appendix-b.html#D. Accessed 5 Feb 2014

[CR16] Dahlui M, Gan DE, Taib NA, Pritam R, Lim J (2012). Predictors of breast cancer screening uptake: a pre intervention community survey in Malaysia. Asian Pac J Cancer Prev APJCP.

[CR17] del Carmen MG, Halpern EF, Kopans DB, Moy B, Moore RH, Goss PE, Hughes KS (2007). Mammographic breast density and race. AJR Am J Roentgenol.

[CR18] DeSantis C, Ma J, Bryan L, Jemal A (2014). Breast cancer statistics, 2013. CA Cancer J Clin.

[CR19] Detsky AS, Laupacis A (2007). Relevance of cost-effectiveness analysis to clinicians and policy makers. JAMA J Am Med Assoc.

[CR20] Detsky AS, Naglie IG (1990). A clinician’s guide to cost-effectiveness analysis. Ann Intern Med.

[CR21] Dinnes J, Moss S, Melia J, Blanks R, Song F, Kleijnen J (2001). Effectiveness and cost-effectiveness of double reading of mammograms in breast cancer screening: findings of a systematic review. Breast.

[CR22] Drukteinis JS, Mooney BP, Flowers CI, Gatenby RA (2013). Beyond mammography: new frontiers in breast cancer screening. Am J Med.

[CR23] Duffy SW, Agbaje O, Tabar L, Vitak B, Bjurstam N, Bjorneld L, Myles JP, Warwick J (2005). Overdiagnosis and overtreatment of breast cancer: estimates of overdiagnosis from two trials of mammographic screening for breast cancer. Breast Cancer Res BCR.

[CR24] Elmore JG, Armstrong K, Lehman CD, Fletcher SW (2005). Screening for breast cancer. JAMA J Am Med Assoc.

[CR25] Fan L, Strasser-Weippl K, Li JJ, St Louis J, Finkelstein DM, Yu KD, Chen WQ, Shao ZM, Goss PE (2014). Breast cancer in China. Lancet Oncol.

[CR26] Ferlay JSI, Ervik M, Dikshit R, Eser S, Mathers C, Rebelo M, Parkin DM, Forman D, Bray, F (2013) GLOBOCAN 2012 v1.0, Cancer Incidence and Mortality Worldwide: IARC CancerBase No. 11 [Internet]. Lyon, France: International Agency for Research on Cancer; 2013. Available from: http://globocan.iarc.fr. Accessed 26 Dec 2013

[CR28] Gershan V, Antevska-Grujoska S (2011). Performance of mammography equipment in the Macedonian breast screening campaign 2008/2009. Radiat Prot Dosim.

[CR29] Goss PE, Lee BL, Badovinac-Crnjevic T, Strasser-Weippl K, Chavarri-Guerra Y, St Louis J, Villarreal-Garza C, Unger-Saldana K, Ferreyra M, Debiasi M, Liedke PE, Touya D, Werutsky G, Higgins M, Fan L, Vasconcelos C, Cazap E, Vallejos C, Mohar A, Knaul F, Arreola H, Batura R, Luciani S, Sullivan R, Finkelstein D, Simon S, Barrios C, Kightlinger R, Gelrud A, Bychkovsky V, Lopes G, Stefani S, Blaya M, Souza FH, Santos FS, Kaemmerer A, de Azambuja E, Zorilla AF, Murillo R, Jeronimo J, Tsu V, Carvalho A, Gil CF, Sternberg C, Duenas-Gonzalez A, Sgroi D, Cuello M, Fresco R, Reis RM, Masera G, Gabus R, Ribeiro R, Knust R, Ismael G, Rosenblatt E, Roth B, Villa L, Solares AL, Leon MX, Torres-Vigil I, Covarrubias-Gomez A, Hernandez A, Bertolino M, Schwartsmann G, Santillana S, Esteva F, Fein L, Mano M, Gomez H, Hurlbert M, Durstine A, Azenha G (2013). Planning cancer control in Latin America and the Caribbean. Lancet Oncol.

[CR30] Goss PE, Strasser-Weippl K, Lee-Bychkovsky BL, Fan L, Li J, Chavarri-Guerra Y, Liedke PE, Pramesh CS, Badovinac-Crnjevic T, Sheikine Y, Chen Z, Qiao YL, Shao Z, Wu YL, Fan D, Chow LW, Wang J, Zhang Q, Yu S, Shen G, He J, Purushotham A, Sullivan R, Badwe R, Banavali SD, Nair R, Kumar L, Parikh P, Subramanian S, Chaturvedi P, Iyer S, Shastri SS, Digumarti R, Soto-Perez-de-Celis E, Adilbay D, Semiglazov V, Orlov S, Kaidarova D, Tsimafeyeu I, Tatishchev S, Danishevskiy KD, Hurlbert M, Vail C, St Louis J, Chan A (2014). Challenges to effective cancer control in China, India, and Russia. Lancet Oncol.

[CR31] Gotzsche PC, Jorgensen KJ (2013). Screening for breast cancer with mammography. Cochrane Database Syst Rev.

[CR32] Harford JB (2011). Breast-cancer early detection in low-income and middle-income countries: do what you can versus one size fits all. Lancet Oncol.

[CR33] Henderson IC, Berry DA, Demetri GD, Cirrincione CT, Goldstein LJ, Martino S, Ingle JN, Cooper MR, Hayes DF, Tkaczuk KH, Fleming G, Holland JF, Duggan DB, Carpenter JT, Frei E, Schilsky RL, Wood WC, Muss HB, Norton L (2003). Improved outcomes from adding sequential Paclitaxel but not from escalating Doxorubicin dose in an adjuvant chemotherapy regimen for patients with node-positive primary breast cancer. J Clin Oncol Off J Am Soc Clin Oncol.

[CR34] Howlader NNA, Krapcho M, Garshell J, Neyman N, Altekruse SF, Kosary CL, Yu M, Ruhl J, Tatalovich Z, Cho H, Mariotto A, Lewis DR, Chen HS, Feuer EJ, Cronin KA (eds) (2013) SEER Cancer Statistics Review, 1975–2010, National Cancer Institute. Bethesda, MD, http://seer.cancer.gov/csr/1975_2010/, based on November 2012 SEER data submission, posted to the SEER web site, April 2013. http://seer.cancer.gov/csr/1975_2010/results_merged/sect_04_breast.pdf

[CR35] Huang CS, Chang KJ, Shen CY (2001). Breast cancer screening in Taiwan and China. Breast Dis.

[CR38] Jorgensen KJ (2013). Mammography screening. Benefits, harms, and informed choice. Dan Med J.

[CR39] Jorgensen KJ, Gotzsche PC (2009). Overdiagnosis in publicly organised mammography screening programmes: systematic review of incidence trends. BMJ.

[CR40] Kadivar M, Joolaee S, Joulaee A, Bahrani N, Hosseini N (2012). Breast cancer knowledge, attitudes and screening behaviors in two groups of Iranian women: physicians and non-health care personnel. J Cancer Educ Off J Am Assoc Cancer Educ.

[CR41] Katz SJ, Hofer TP (1994). Socioeconomic disparities in preventive care persist despite universal coverage. Breast and cervical cancer screening in Ontario and the United States. JAMA J Am Med Assoc.

[CR42] Kerlikowske K, Grady D, Rubin SM, Sandrock C, Ernster VL (1995). Efficacy of screening mammography. A meta-analysis. JAMA J Am Med Assoc.

[CR43] Laxminarayan R, Chow J, Shahid-Salles SA (2006) Intervention Cost-Effectiveness: Overview of Main Messages. In: Jamison DT, Breman JG, Measham AR et al. (eds) Disease Control Priorities in Developing Countries, 2nd edn. Washington (DC) 21250358

[CR44] Lee BL, Liedke PE, Barrios CH, Simon SD, Finkelstein DM, Goss PE (2012). Breast cancer in Brazil: present status and future goals. Lancet Oncol.

[CR45] Liberato NL, Marchetti M, Barosi G (2007). Cost effectiveness of adjuvant trastuzumab in human epidermal growth factor receptor 2-positive breast cancer. J Clin Oncol Off J Am Soc Clin Oncol.

[CR46] Mandelson MT, Oestreicher N, Porter PL, White D, Finder CA, Taplin SH, White E (2000). Breast density as a predictor of mammographic detection: comparison of interval- and screen-detected cancers. J Natl Cancer Inst.

[CR47] Maree J, Wright S, Lu X (2013). Breast cancer risks and screening practices among women living in a resource poor community in Tshwane, South Africa. Breast J.

[CR48] Marmot MG (2013). Sorting through the arguments on breast screening. JAMA J Am Med Assoc.

[CR6] Marmot MG, Altman DG, Cameron DA, Dewar JA, Thompson SG, Wilcox M (2012). The benefits and harms of breast cancer screening: an independent review. Lancet.

[CR49] Matkowski R, Szynglarewicz B (2011). First report of introducing population-based breast cancer screening in Poland: experience of the 3-million population region of Lower Silesia. Cancer Epidemiol.

[CR50] Mauad EC, Silva TB, Haikel RL, Bauab S, Longatto-Filho A (2011). Is community intervention in breast cancer screening in Brazil feasible?. J Med Screen.

[CR51] Melnikow J, Tancredi DJ, Yang Z, Ritley D, Jiang Y, Slee C, Popova S, Rylett P, Knutson K, Smalley S (2013). Program-specific cost-effectiveness analysis: breast cancer screening policies for a safety-net program. Value Health J Int Soc Pharmacoecon Outcomes Res.

[CR52] Mo M, Liu GY, Zheng Y, Di LF, Ji YJ, Lv LL, Chen YY, Peng WJ, Zhu JR, Bao PP, Ding JH, Chang C, Luo JF, Cao ZG, Xu WH, Shao ZM (2013). Performance of breast cancer screening methods and modality among Chinese women: a report from a society-based breast screening program (SBSP) in Shanghai. Springerplus.

[CR53] Moore MA, Tajima K, Anh PH, Aydemir G, Basu PS, Bhurgri Y, Chen K, Gajalakshmi V, Hirose K, Jarrahi AM, le Ngoan T, Qiao YL, Shin HR, Sriamporn S, Srivatanakul P, Tokudome S, Yoo KY, Tsuda H (2003). Grand challenges in global health and the practical prevention program? Asian focus on cancer prevention in females of the developing world. Asian Pac J Cancer Prev APJCP.

[CR54] National Breast Cancer Foundation (2014) Mammogram. http://www.nationalbreastcancer.org/diagnostic-mammogram. Accessed 2 April 2014

[CR55] National Institute for Health and Care Excellence (2014) Measuring effectiveness and cost effectiveness: the QALY. http://www.nice.org.uk/newsroom/features/measuringeffectivenessandcosteffectivenesstheqaly.jsp. Accessed 05 April 2014

[CR56] Okonkwo QL, Draisma G, der Kinderen A, Brown ML, de Koning HJ (2008). Breast cancer screening policies in developing countries: a cost-effectiveness analysis for India. J Natl Cancer Inst.

[CR57] Pace LE, Keating NL (2014). A systematic assessment of benefits and risks to guide breast cancer screening decisions. JAMA J Am Med Assoc.

[CR58] Paci E (2012). Summary of the evidence of breast cancer service screening outcomes in Europe and first estimate of the benefit and harm balance sheet. J Med Screen.

[CR59] Peregrino AA, Vianna CM, de Almeida CE, Gonzales GB, Machado SC, Costa e Silva FV, Rodrigues MP (2012). Analysis of Cost-effectiveness of screening for breast cancer with conventional mammography, digital and magnetic resonance imaging. Cienc Saude Coletiva.

[CR60] Pharoah PD, Sewell B, Fitzsimmons D, Bennett HS, Pashayan N (2013). Cost effectiveness of the NHS breast screening programme: life table model. BMJ.

[CR62] Puliti D, Duffy SW, Miccinesi G, de Koning H, Lynge E, Zappa M, Paci E (2012). Overdiagnosis in mammographic screening for breast cancer in Europe: a literature review. J Med Screen.

[CR63] Quanstrum KH, Hayward RA (2010). Lessons from the mammography wars. N Engl J Med.

[CR64] Remak E, Brazil L (2004). Cost of managing women presenting with stage IV breast cancer in the United Kingdom. Br J Cancer.

[CR65] Rodriguez-Cuevas S, Guisa-Hohenstein F, Labastida-Almendaro S (2009). First breast cancer mammography screening program in Mexico: initial results 2005–2006. Breast J.

[CR66] Salas D, Ibanez J, Roman R, Cuevas D, Sala M, Ascunce N, Zubizarreta R, Castells X (2011). Effect of start age of breast cancer screening mammography on the risk of false-positive results. Prev Med.

[CR67] Sarvazyan A, Egorov V, Son JS, Kaufman CS (2008). Cost-effective screening for breast cancer worldwide: current state and future directions. Breast Cancer Basic Clin Res.

[CR68] Sato M, Kawai M, Nishino Y, Shibuya D, Ohuchi N, Ishibashi T (2012). Cost-effectiveness analysis for breast cancer screening: double reading versus single + CAD reading. Breast Cancer.

[CR70] Silva TB, Mauad EC, Carvalho AL, Jacobs LA, Shulman LN (2013). Difficulties in implementing an organized screening program for breast cancer in Brazil with emphasis on diagnostic methods. Rural Remote Health.

[CR71] Smith RA, Saslow D, Sawyer KA, Burke W, Costanza ME, Evans WP, Foster RS, Hendrick E, Eyre HJ, Sener S (2003). American Cancer Society guidelines for breast cancer screening: update 2003. CA Cancer J Clin.

[CR72] Stamenic V, Strnad M (2011). Urban-rural differences in a population-based breast cancer screening program in Croatia. Croat Med J.

[CR73] Tatla RK, Paszat LF, Bondy SJ, Chen Z, Chiarelli AM, Mai V (2003). Socioeconomic status and returning for a second screen in the Ontario breast screening program. Breast.

[CR36] The International Agency for Research on Cancer (IARC) (2002) Handbook of Cancer Prevention, vol 7. http://www.iarc.fr/en/publications/pdfs-online/prev/handbook7/Handbook7_Breast.pdf. Accessed 12 Feb 2014

[CR37] The International Agency for Research on Cancer (IARC) (2013) Latest World Cancer Statistics. Press Release No 223. http://www.iarc.fr/en/media-centre/pr/2013/pdfs/pr223_E.pdf. Accessed 20 Jan 2014

[CR83] The World Bank (2014a) New Country Classifications. http://data.worldbank.org/news/new-country-classifications. Accessed 15 Jan 2014

[CR02] The World Bank (2014b) Topics. Indicators: Population; Population Density; Life expectancy at birth, female. http://data.worldbank.org/topic. Accessed 15 Mar 2014

[CR74] Tiezzi DG (2013). Breast cancer screening in Brazil: there is still time to rethink. Revista brasileira de ginecologia e obstetricia: revista da Federacao Brasileira das Sociedades de Ginecologia e Obstetricia.

[CR75] Tonelli M, Connor Gorber S, Joffres M, Dickinson J, Singh H, Lewin G, Birtwhistle R, Fitzpatrick-Lewis D, Hodgson N, Ciliska D, Gauld M, Liu YY (2011). Recommendations on screening for breast cancer in average-risk women aged 40–74 years. CMAJ Can Med Assoc J (journal de l’Association medicale canadienne).

[CR27] United Kingdom Co-ordinating Committee on Cancer Research (2002). The frequency of breast cancer screening: results from the UKCCCR Randomised Trial. Eur J Cancer.

[CR69] US Preventive Services Task Force (2009) Screening for breast cancer: U.S. Preventive Services Task Force recommendation statement. Ann Internal Med 151(10):716–726, W-236. doi:10.7326/0003-4819-151-10-200911170-00008 10.7326/0003-4819-151-10-200911170-00008 19920272

[CR76] van Ineveld BM, van Oortmarssen GJ, de Koning HJ, Boer R, van der Maas PJ (1993). How cost-effective is breast cancer screening in different EC countries?. Eur J Cancer.

[CR77] Voevodina O, Billich C, Arand B, Nagel G (2013). Association of Mediterranean diet, dietary supplements and alcohol consumption with breast density among women in South Germany: a cross-sectional study. BMC Public Health.

[CR78] Wald NJ, Murphy P, Major P, Parkes C, Townsend J, Frost C (1995). UKCCCR multicentre randomised controlled trial of one and two view mammography in breast cancer screening. BMJ.

[CR79] Weinstein MC, Stason WB (1977). Foundations of cost-effectiveness analysis for health and medical practices. N Engl J Med.

[CR80] Welch HG, Black WC (2010). Overdiagnosis in cancer. J Natl Cancer Inst.

[CR81] WHO (2010) Baseline medical survey on medical devices. http://phstwlp2.partners.org:2665/hq/2011/WHO_HSS_EHT_DIM_11.01_eng.pdf (2011). Accessed 19 Mar 2014

[CR82] WHO (2008) Guide for effective programmes; cancer control: knowledge into action; module 4: palliative care. Geneva: World Health Organization. http://www.who.int/cancer/modules/en/index.html. Accessed 20 Jan 2014

[CR84] Wubker A (2013). Explaining variations in breast cancer screening across European countries. Eur J Health Econ HEPAC Health Econ Prev Care.

[CR85] Yip CH, Smith RA, Anderson BO, Miller AB, Thomas DB, Ang ES, Caffarella RS, Corbex M, Kreps GL, McTiernan A (2008). Guideline implementation for breast healthcare in low- and middle-income countries: early detection resource allocation. Cancer.

[CR86] Yoo KB, Kwon JA, Cho E, Kang MH, Nam JM, Choi KS, Kim EK, Choi YJ, Park EC (2013). Is mammography for breast cancer screening cost-effective in both Western and asian countries? Results of a systematic review. Asian Pac J Cancer Prev APJCP.

[CR87] Zakharova N, Duffy S, Mackay J, Kotlyarov E (2011). The introduction of a breast cancer screening programme in a region with a population at medium risk for developing breast cancer: Khanty-Mansiysky autonomous Okrug-Ugra (Russian Federation). Ecancermedicalscience.

[CR88] Zakharova NA (2013). Experience in the implementation of screening program for early detection of breast cancer in the Khanty-Mansi Autonomous Region-Yugra. Vopr Onkol.

